# 1-(Thio­phen-2-yl)ethanone thio­semi­carbazone

**DOI:** 10.1107/S1600536811011986

**Published:** 2011-04-16

**Authors:** Papa Aly Gaye, Adama Sy, Aminata Diassé Sarr, Mohamed Gaye, Celine Besnard

**Affiliations:** a Département de Chimie, Faculté des Sciences et Techniques, Université Cheikh Anta Diop, Dakar, Senegal; b Laboratoire de Cristallochimie, Ecole de Physique, 24 Quai Ernest-Ansermet, 1211 Gebeve, Switzerland

## Abstract

The title compound, C_7_H_9_N_3_S_2_, crystallizes with two unique mol­ecules in the unit cell, both present as thio­semicarbazide tautomers. The mol­ecules differ principally in the dihedral angles between the thio­phene ring planes and the planes through the non-H atoms of the hydrazinecarbothio­amide units, *viz.* 9.80 (8)° for one mol­ecule and 19.37 (7)° for the other. The hydrazinecarbothio­amide units are reasonably planar, with r.m.s. deviations of 0.001Å for each of the mol­ecules. In the crystal, N—H⋯S hydrogen bonds link like mol­ecules into *R*_2_^2^(8) inversion dimers. A three-dimensional network structure is generated by additional N—H⋯S hydrogen bonds and weak C—H⋯S contacts between the unique mol­ecules.

## Related literature

For related structures, see: Avsar *et al.* (2003 ▶); Arslan *et al.* (2004 ▶); Kusaï *et al.* (2009 ▶). For graph-set motifs, see: Bernstein *et al.* (1995 ▶). For the weighting scheme, see: Prince (1982 ▶); Watkin (1994 ▶).
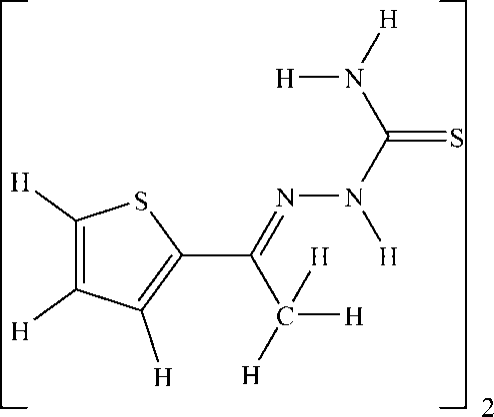


## Experimental

### 

#### Crystal data

C_7_H_9_N_3_S_2_
*M* *_r_* = 199.31
Triclinic, 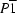

*a* = 9.0037 (9) Å
*b* = 9.7800 (9) Å
*c* = 12.1428 (12) Å
α = 104.575 (8)°
β = 103.345 (8)°
γ = 108.227 (8)°
*V* = 925.52 (18) Å^3^
*Z* = 4
Mo *K*α radiation
μ = 0.52 mm^−1^
*T* = 140 K
0.50 × 0.40 × 0.30 mm

#### Data collection

Stoe IPDS diffractometer
13175 measured reflections
5386 independent reflections
4634 reflections with *I* > 2σ(*I*)
*R*_int_ = 0.033

#### Refinement

*R*[*F* ^2^ > 2σ(*F* ^2^)] = 0.034
*wR*(*F* ^2^) = 0.053
*S* = 1.00
5373 reflections
217 parameters
H-atom parameters constrained
Δρ_max_ = 0.40 e Å^−3^
Δρ_min_ = −0.39 e Å^−3^

### 

Data collection: *IPDS* (Stoe & Cie, 1996 ▶); cell refinement: *IPDS*; data reduction: *X-RED* (Stoe & Cie 1996 ▶); program(s) used to solve structure: *SUPERFLIP* (Palatinus & Chapuis, 2007 ▶); program(s) used to refine structure: *CRYSTALS* (Betteridge *et al.*, 2003 ▶); molecular graphics: *CAMERON* (Watkin *et al.*, 1996 ▶); software used to prepare material for publication: *CRYSTALS*.

## Supplementary Material

Crystal structure: contains datablocks global, I. DOI: 10.1107/S1600536811011986/pb2061sup1.cif

Structure factors: contains datablocks I. DOI: 10.1107/S1600536811011986/pb2061Isup2.hkl

Additional supplementary materials:  crystallographic information; 3D view; checkCIF report

## Figures and Tables

**Table 1:** Hydrogen-bond geometry (Å, °)

*D*—H⋯*A*	*D*—H	H⋯*A*	*D*⋯*A*	*D*—H⋯*A*
N1—H11⋯S3^i^	0.83	2.56	3.3609 (16)	162
N21—H212⋯S3^ii^	0.83	2.66	3.4691 (16)	167
N24—H241⋯S23^iii^	0.85	2.77	3.6128 (14)	174
C7—H72⋯S23^ii^	0.98	2.83	3.7236 (16)	153

Symmetry codes: (i) 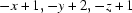; (ii) 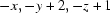; (iii) 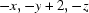.

**Fig. 1.**
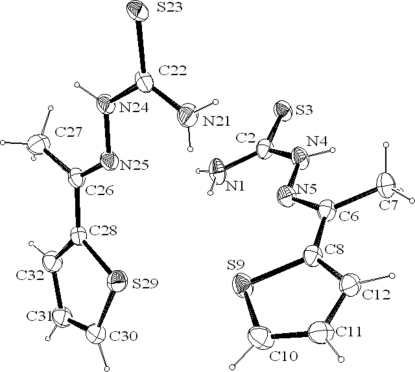
The asymmetric unit of the title compound, showing the atom-numbering scheme. Displacement ellipsoids are drawn at the 50% probability level.

**Fig. 2.**
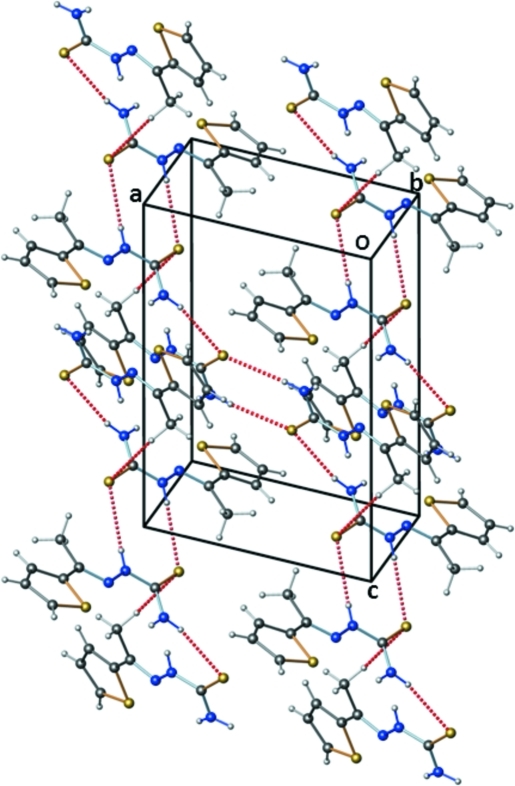
Crystal packing of 1 viewed down the b axis. Hydrogen bonds are drawn as dashed lines.

## supplementary crystallographic information

### Comment

NMR analysis of the title compound, (C_7_H_9_N_3_S_2_)_2_, (see experimental) shows that two forms are present in solution with the thiosemicarbazide moiety being partially transformed into the thioenolsemicarbazide. The X-ray structure determination reveals that the compound crystallizes in the triclinic space group P-1 with two molecules in the asymmetric unit, both of which are present as the thiosemicarbazide tautomer. The molecular geometry is illustrated in Fig. 1. The C—S bond lengths 1.6921 (15) Å and 1.6884 (15) Å confirm the double bond character and are comparable to those observed for 1-(biphenyl-4-carbonyl)-3-*p*-tolyl-thiourea [1.647 (3) Å for C—S, 1.217 (3) and 1.224 (3) Å] (Avsar *et al.*, 2003). The C—N bond lengths are in the range [1.2920 (17) - 1.4122 (16) Å] which are shorter than normal single C—N bond lengths (Arslan *et al.*, 2004).

Both unique molecules form inversion related dimers with R^2^_2_(8) graph-set motifs (Bernstein *et al.*, 1995) through N1—H11···S3 and N24—H241···S23 hydrogen bonds. Unique molecules are further linked by N21—H212···S3 bonds supported by a weak C7—H72···S3 contacts which generate additional centrosymmetric R^2^_2_(11) motifs and a three dimensional network (Fig. 2).

### Experimental

2-Acetyl thiophene (1,2618 g, 10 mmol) was reacted with thiosemicarbazide (0.9114 g, 10 mmol) in CH_3_OH (50 ml) solution, to give the corresponding compound after one hour under reflux. After cooling to room temperature, a yellow solid was isolated and washed  twice with diethyl ether. Yield: 79.5%. m.p. 142–146 °C. Anal. Calc. for C_7_H_9_N_3_S_2_ (%): C, 42.19; H, 4.55; N, 21.08. Found: C, 42.22; H, 4.53; N, 21.01. Selected IR data (cm^-1^, KBr pellet): 3450, 3250 (ν NH), 1630 (ν C=N), 1160 (ν C=S). ^1^H NMR (200 MHz, CD_6_Cl, δ, p.p.m.): 2.32 (s, 3H, –CH_3_); 7.42 (s, 2H, –NH_2_); 8.32 (s, 1H, –NH); 7.07–7.58 (m, 3H, C_4_H_3_S); 10.36 (s, 1H, SH);. ^13^C NMR (200 MHz, CD_3_Cl, δ, p.p.m.): 14.01 (–CH_3_); 68.09 (O—CH~2~); 70.12 (O– CH~2~); 127.12–143.152 (C_4_H_3_S); 145.30 (C=N); 178.02 (C=S). A CH_3_Cl solution of the title compound was mixed with ethanol (1/1). After several days, colorless block-shaped single crystals suitable for X-ray crystallographic analysis were obtained.

### Refinement

The H atoms were all located in a difference map, but those attached to carbon atoms were repositioned geometrically. The H atoms were initially refined with soft restraints on the bond lengths and angles to regularize their geometry (C—H in the range 0.93–0.98, N—H in the range 0.86–0.89 N—H to 0.86 O—H = 0.82 Å) and *U*_iso_(H) (in the range 1.2–1.5 times *U*_eq_ of the parent atom), after which the positions were refined with riding constraints.

### Crystal data

**Table:**

C_7_H_9_N_3_S_2_	*Z* = 4
*M**_r_* = 199.31	*F*(000) = 416
Triclinic, *P*1	*D*_x_ = 1.430 Mg m^−^^3^
Hall symbol: -P 1	Mo *K*α radiation, λ = 0.71073 Å
*a* = 9.0037 (9) Å	Cell parameters from 0 reflections
*b* = 9.7800 (9) Å	θ = 0–0°
*c* = 12.1428 (12) Å	µ = 0.52 mm^−^^1^
α = 104.575 (8)°	*T* = 140 K
β = 103.345 (8)°	Parallelepiped, yellow
γ = 108.227 (8)°	0.50 × 0.40 × 0.30 mm
*V* = 925.52 (18) Å^3^	

### Data collection

**Table:**

Stoe IPDS diffractometer	*R*_int_ = 0.033
graphite	θ_max_ = 30.9°, θ_min_ = 2.3°
ω scans	*h* = −12→12
13175 measured reflections	*k* = −12→13
5386 independent reflections	*l* = −17→17
4634 reflections with *I* > 2σ(*I*)	

### Refinement

**Table:**

Refinement on *F*^2^	Primary atom site location: structure-invariant direct methods
Least-squares matrix: full	Hydrogen site location: inferred from neighbouring sites
*R*[*F*^2^ > 2σ(*F*^2^)] = 0.034	H-atom parameters constrained
*wR*(*F*^2^) = 0.053	Method, part 1, Chebychev polynomial, (Watkin, 1994, *P*rince, 1982) [*w*eight] = 1.0/[A_0_*T_0_(x) + A_1_*T_1_(x) ··· + A_n-1_]*T_n-1_(x)] where A_i_ are the Chebychev coefficients listed belo*w* and x = *F* /*F*max Method = Robust Weighting (*P*rince, 1982) W = [*w*eight] * [1-(delta*F*/6*sigma*F*)^2^]^2^ A_i_ are: 17.0 19.0 6.62
*S* = 1.00	(Δ/σ)_max_ = 0.001
5373 reflections	Δρ_max_ = 0.40 e Å^−^^3^
217 parameters	Δρ_min_ = −0.39 e Å^−^^3^
0 restraints	

### Special details

**Table:**

Experimental. Reflections affected by the beam-stop were not included in the refinement.

### Fractional atomic coordinates and isotropic or equivalent isotropic displacement parameters (Å^2^)

**Table:**

	*x*	*y*	*z*	*U*_iso_*/*U*_eq_	
N1	0.26295 (15)	0.85450 (15)	0.42449 (11)	0.0287	
C2	0.23744 (17)	0.93285 (17)	0.51896 (12)	0.0224	
S3	0.37191 (5)	1.11020 (4)	0.61614 (4)	0.0264	
N4	0.09273 (14)	0.86531 (14)	0.53590 (11)	0.0255	
N5	−0.00908 (14)	0.71581 (14)	0.46373 (11)	0.0234	
C6	−0.13491 (16)	0.65062 (17)	0.49325 (12)	0.0217	
C7	−0.17662 (18)	0.72896 (18)	0.59706 (13)	0.0276	
C8	−0.23815 (16)	0.49066 (17)	0.41921 (13)	0.0222	
S9	−0.19349 (5)	0.39888 (5)	0.29754 (4)	0.0286	
C10	−0.35441 (19)	0.22919 (18)	0.26747 (15)	0.0313	
C11	−0.43840 (19)	0.24169 (18)	0.34678 (15)	0.0295	
C12	−0.37117 (18)	0.39098 (17)	0.43411 (14)	0.0260	
N21	−0.13026 (16)	0.79416 (16)	0.21760 (12)	0.0317	
C22	−0.07537 (17)	0.87345 (17)	0.15117 (12)	0.0240	
S23	−0.15386 (5)	0.99727 (5)	0.11278 (4)	0.0283	
N24	0.05087 (15)	0.85422 (14)	0.11585 (11)	0.0245	
N25	0.10126 (15)	0.74112 (14)	0.13786 (10)	0.0233	
C26	0.22938 (17)	0.73180 (16)	0.11099 (12)	0.0213	
C27	0.33339 (19)	0.83708 (19)	0.06322 (15)	0.0311	
C28	0.27075 (17)	0.60356 (16)	0.12664 (12)	0.0210	
S29	0.14366 (5)	0.46442 (5)	0.16514 (4)	0.0294	
C30	0.2690 (2)	0.36424 (19)	0.15960 (14)	0.0324	
C31	0.4021 (2)	0.43291 (19)	0.13032 (15)	0.0314	
C32	0.40373 (19)	0.57103 (18)	0.11115 (14)	0.0282	
H71	−0.2700	0.6650	0.6064	0.0418*	
H73	−0.1962	0.8165	0.5899	0.0414*	
H72	−0.0819	0.7680	0.6714	0.0395*	
H101	−0.3781	0.1398	0.2028	0.0387*	
H111	−0.5275	0.1609	0.3456	0.0342*	
H121	−0.4131	0.4187	0.4973	0.0309*	
H271	0.4474	0.8480	0.0869	0.0485*	
H273	0.3333	0.9390	0.0904	0.0470*	
H272	0.2894	0.8001	−0.0250	0.0484*	
H301	0.2423	0.2727	0.1735	0.0391*	
H311	0.4849	0.3958	0.1244	0.0388*	
H321	0.4848	0.6364	0.0904	0.0338*	
H41	0.0815	0.9026	0.6019	0.0329*	
H241	0.0787	0.8967	0.0667	0.0309*	
H12	0.1936	0.7641	0.3821	0.0374*	
H211	−0.0889	0.7344	0.2366	0.0407*	
H11	0.3561	0.8847	0.4174	0.0369*	
H212	−0.2011	0.8090	0.2464	0.0402*	

### Atomic displacement parameters (Å^2^)

**Table:**

	*U*^11^	*U*^22^	*U*^33^	*U*^12^	*U*^13^	*U*^23^
N1	0.0205 (6)	0.0368 (7)	0.0220 (6)	0.0057 (5)	0.0103 (5)	0.0038 (5)
C2	0.0188 (6)	0.0292 (7)	0.0221 (6)	0.0107 (5)	0.0084 (5)	0.0108 (6)
S3	0.02314 (17)	0.02531 (17)	0.03061 (19)	0.00811 (14)	0.01403 (15)	0.00688 (15)
N4	0.0206 (6)	0.0279 (6)	0.0274 (6)	0.0080 (5)	0.0136 (5)	0.0058 (5)
N5	0.0191 (5)	0.0264 (6)	0.0245 (6)	0.0089 (5)	0.0085 (5)	0.0075 (5)
C6	0.0174 (6)	0.0298 (7)	0.0221 (6)	0.0116 (5)	0.0084 (5)	0.0114 (6)
C7	0.0236 (7)	0.0346 (8)	0.0257 (7)	0.0107 (6)	0.0129 (6)	0.0086 (6)
C8	0.0185 (6)	0.0293 (7)	0.0238 (7)	0.0125 (5)	0.0093 (5)	0.0115 (6)
S9	0.02536 (18)	0.03112 (19)	0.03085 (19)	0.01106 (15)	0.01547 (15)	0.00804 (15)
C10	0.0276 (7)	0.0267 (7)	0.0396 (9)	0.0120 (6)	0.0135 (7)	0.0079 (7)
C11	0.0253 (7)	0.0270 (7)	0.0417 (9)	0.0122 (6)	0.0152 (7)	0.0148 (7)
C12	0.0225 (7)	0.0309 (7)	0.0327 (8)	0.0136 (6)	0.0150 (6)	0.0152 (6)
N21	0.0333 (7)	0.0477 (8)	0.0356 (7)	0.0287 (6)	0.0220 (6)	0.0234 (6)
C22	0.0235 (7)	0.0300 (7)	0.0188 (6)	0.0144 (6)	0.0068 (5)	0.0044 (5)
S23	0.03127 (19)	0.0329 (2)	0.03109 (19)	0.02194 (16)	0.01527 (16)	0.01188 (16)
N24	0.0271 (6)	0.0323 (6)	0.0242 (6)	0.0197 (5)	0.0130 (5)	0.0119 (5)
N25	0.0249 (6)	0.0293 (6)	0.0210 (6)	0.0165 (5)	0.0091 (5)	0.0088 (5)
C26	0.0201 (6)	0.0269 (7)	0.0170 (6)	0.0114 (5)	0.0052 (5)	0.0059 (5)
C27	0.0293 (8)	0.0347 (8)	0.0405 (9)	0.0175 (7)	0.0173 (7)	0.0198 (7)
C28	0.0209 (6)	0.0259 (7)	0.0173 (6)	0.0109 (5)	0.0071 (5)	0.0066 (5)
S29	0.03128 (19)	0.0318 (2)	0.0328 (2)	0.01480 (16)	0.01744 (16)	0.01445 (16)
C30	0.0462 (9)	0.0327 (8)	0.0278 (7)	0.0228 (7)	0.0162 (7)	0.0134 (6)
C31	0.0376 (8)	0.0386 (9)	0.0317 (8)	0.0266 (7)	0.0163 (7)	0.0154 (7)
C32	0.0264 (7)	0.0351 (8)	0.0305 (8)	0.0177 (6)	0.0134 (6)	0.0128 (6)

### Geometric parameters (Å, °)

**Table:**

N1—C2	1.3210 (17)	N21—C22	1.3249 (19)
N1—H12	0.845	N21—H211	0.836
N1—H11	0.831	N21—H212	0.828
C2—S3	1.6921 (15)	C22—S23	1.6884 (15)
C2—N4	1.3528 (17)	C22—N24	1.3525 (17)
N4—N5	1.3754 (17)	N24—N25	1.3811 (16)
N4—H41	0.836	N24—H241	0.849
N5—C6	1.2920 (17)	N25—C26	1.2923 (17)
C6—C7	1.4931 (19)	C26—C27	1.491 (2)
C6—C8	1.455 (2)	C26—C28	1.4586 (19)
C7—H71	0.928	C27—H271	0.963
C7—H73	0.947	C27—H273	0.969
C7—H72	0.978	C27—H272	0.977
C8—S9	1.7270 (14)	C28—S29	1.7243 (14)
C8—C12	1.3696 (19)	C28—C32	1.3719 (18)
S9—C10	1.7127 (16)	S29—C30	1.7122 (16)
C10—C11	1.360 (2)	C30—C31	1.351 (2)
C10—H101	0.941	C30—H301	0.921
C11—C12	1.412 (2)	C31—C32	1.424 (2)
C11—H111	0.927	C31—H311	0.935
C12—H121	0.948	C32—H321	0.938
			
C2—N1—H12	119.3	C22—N21—H211	121.3
C2—N1—H11	119.5	C22—N21—H212	120.1
H12—N1—H11	119.4	H211—N21—H212	118.3
N1—C2—S3	124.09 (11)	N21—C22—S23	122.70 (11)
N1—C2—N4	116.62 (13)	N21—C22—N24	117.28 (13)
S3—C2—N4	119.28 (10)	S23—C22—N24	120.01 (11)
C2—N4—N5	119.09 (12)	C22—N24—N25	118.70 (12)
C2—N4—H41	118.7	C22—N24—H241	117.3
N5—N4—H41	119.2	N25—N24—H241	122.2
N4—N5—C6	116.53 (12)	N24—N25—C26	117.62 (12)
N5—C6—C7	124.02 (13)	N25—C26—C27	125.81 (13)
N5—C6—C8	116.11 (12)	N25—C26—C28	115.93 (13)
C7—C6—C8	119.87 (12)	C27—C26—C28	118.24 (12)
C6—C7—H71	112.8	C26—C27—H271	112.9
C6—C7—H73	112.7	C26—C27—H273	111.6
H71—C7—H73	107.0	H271—C27—H273	107.4
C6—C7—H72	109.4	C26—C27—H272	110.7
H71—C7—H72	109.4	H271—C27—H272	107.9
H73—C7—H72	105.1	H273—C27—H272	106.0
C6—C8—S9	120.53 (10)	C26—C28—S29	121.16 (10)
C6—C8—C12	128.75 (13)	C26—C28—C32	128.13 (13)
S9—C8—C12	110.66 (11)	S29—C28—C32	110.68 (11)
C8—S9—C10	91.72 (8)	C28—S29—C30	91.80 (8)
S9—C10—C11	112.09 (12)	S29—C30—C31	112.44 (12)
S9—C10—H101	121.9	S29—C30—H301	121.1
C11—C10—H101	126.1	C31—C30—H301	126.4
C10—C11—C12	112.29 (14)	C30—C31—C32	112.12 (14)
C10—C11—H111	124.1	C30—C31—H311	124.5
C12—C11—H111	123.6	C32—C31—H311	123.4
C11—C12—C8	113.24 (13)	C31—C32—C28	112.97 (14)
C11—C12—H121	123.2	C31—C32—H321	126.0
C8—C12—H121	123.5	C28—C32—H321	121.0

### Hydrogen-bond geometry (Å, °)

**Table:**

*D*—H···*A*	*D*—H	H···*A*	*D*···*A*	*D*—H···*A*
N1—H11···S3^i^	0.83	2.56	3.3609 (16)	162
N21—H212···S3^ii^	0.83	2.66	3.4691 (16)	167
N24—H241···S23^iii^	0.85	2.77	3.6128 (14)	174
C7—H72···S23^ii^	0.98	2.83	3.7236 (16)	153

Symmetry codes: (i) −*x*+1, −*y*+2, −*z*+1; (ii) −*x*, −*y*+2, −*z*+1; (iii) −*x*, −*y*+2, −*z*.
